# Potential of Progressive and Disruptive Innovation-Driven Cost Reductions of Green Hydrogen Production

**DOI:** 10.1021/acs.energyfuels.4c01247

**Published:** 2024-05-18

**Authors:** Thorin Daniel, Lei Xing, Qiong Cai, Lirong Liu, Jin Xuan

**Affiliations:** † School of Chemistry and Chemical Engineering, University of Surrey, Guildford GU2 7XH, U.K.; ‡ Centre for Environment and Sustainability, University of Surrey, Guildford GU2 7XH, U.K.

## Abstract

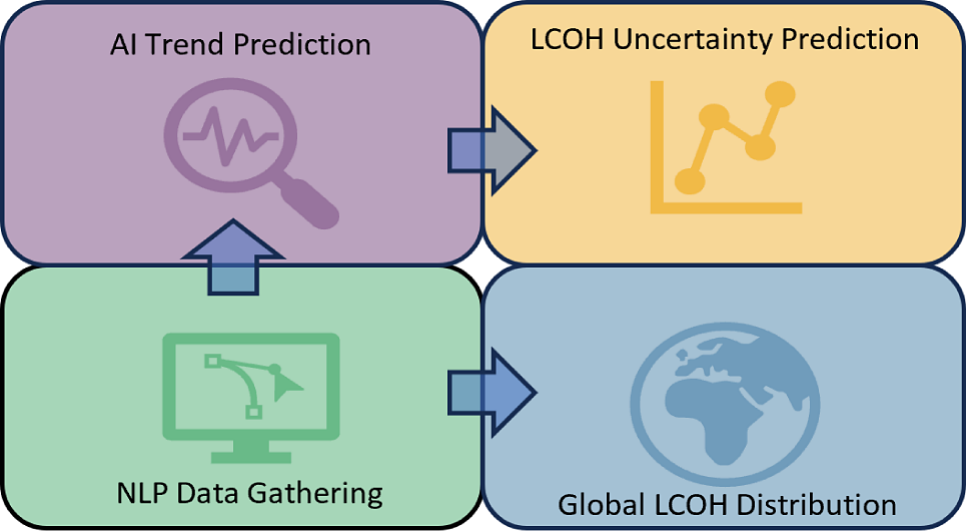

Green hydrogen from water electrolysis is a key driver for energy and industrial decarbonization. The prediction of the future green hydrogen cost reduction is required for investment and policy-making purposes but is complicated due to a lack of data, incomplete accounting for costs, and difficulty justifying trend predictions. A new AI-assisted data-driven prediction model is developed for an in-depth analysis of the current and future levelized costs of green hydrogen, driven by both progressive and disruptive innovations. The model uses natural language processing to gather data and generate trends for the technological development of key aspects of electrolyzer technology. Through an uncertainty analysis, green hydrogen costs have been shown to likely reach the key target of <$2.5 kg^–1^ by 2030 via progressive innovations, and beyond this point, disruptive technological developments are required to affect significantly further decease cost. Additionally, the global distribution of green hydrogen costs has been calculated. This work creates a comprehensive analysis of the levelized cost of green hydrogen, including the important balance of plant components, both now and as electrolyzer technology develops, and offers a likely prediction for how the costs will develop over time.

## 1 Introduction

To meet various climate and emission targets, renewably powered water electrolysis-generated hydrogen, widely known as green hydrogen, is crucial to the decarbonization of several major sectors. It also plays important roles in bridging the gap between intermittent renewable energy sources and constant energy demand.^1−3^ In order to bring green hydrogen into competition with traditional carbon-intensive hydrogen (known as gray hydrogen), its price must decrease below $2.5 kg^–1^, with a longer-term target of $1 kg^–1^ in place to completely supplant gray hydrogen.^4,5^ A great deal of uncertainty surrounds the current and future levelized costs of green hydrogen due to the relative immaturity of electrolyzer technologies. However, accurate cost prediction is needed to guide technology development and drive investment.

Green hydrogen production is reliant on expensive electrolysis technologies that need further development in order to lower costs.^6^ As an emerging technology, its cost reduction will be reliant on both progressive and disruptive innovations. Progressive technological development is the continuous and gradual improvement of a technology over time, so that year on year, a technology trends toward higher cost-effectiveness. Meanwhile, disruptive technological development is when a new technology is able to supplant a different or older technology by being a significant improvement or step change.^7^ In the field of green hydrogen production, progressive innovation is important to continuous hydrogen cost reductions, but it may also result in a cost plateau as further innovation becomes cost prohibitive or too incremental. Whereas disruptive innovation relies on more immature technology, which is inherently less likely to succeed but could result in a significant step change impact on cost reduction.

The current wide range of levelized cost of hydrogen (LCOH) reported by literature, $3–6 kg^–1^, illustrates the difficulty of providing an accurate estimation of green hydrogen production costs’ trends over the next few years.^4^ Progressive technological development has been studied by Vartiainen et al.,^6^ where the LCOH for discrete years has been determined using decreasing technology costs. Similar analysis of several electrolyzer technologies occurred in an analysis by George et al.,^8^ and resulted in high LCOH predictions compared to predictions by IRENA and the IEA.^4,9^ Disruptive technologies have been analyzed by Yang et al.^10^ and Parnamae et al.,^11^ where anion and bipolar technologies were analyzed but limited LCOH information was available due to the immaturity of the technologies. Vartiainen et al.^6^ included an area of uncertainty for technological development but did not offer a means of describing where within the region there is a higher probability. Figure S8 displays the literature data comparisons.

One common limitation of previous studies on green hydrogen cost analysis is the lack of high-quality Balance of Plant (BOP) analysis. BOP units contribute a huge amount to the total hydrogen cost, and their inclusion or noninclusion greatly affects the comparability and accuracy of the LCOH. The BOP costs include those from the water purifier, compressor, pressure swing adsorption (PSA), thermal management systems, pumps, piping, and control. The majority of literature focuses largely or solely on the electrolyzer cell’s main components and assigns a factor to account for the balance of the cell and the balance of plant.^12,13^ This can result in untrue assumptions if a cost making up the total has high variability. For example, if the cost of the BOP is assumed to be a fixed proportion of the cost of the electrolyzer, but the electrolyzer cost is projected to decrease over time, this would cause the BOP costs to fall arbitrarily, which may be erroneous.

The prediction of the trend of future hydrogen production costs requires large amounts of data from the literature. However, previous studies have been limited by the difficulties and time commitment required for data processing. The use of AI models in research has allowed the development of tools to speed the efficiency and quality of data collection, such as work by Macêdo et al.,^14^ where a bidirectional encoder representation from transformers (BERT) natural language processing (NLP) model was used to capture raw data and generate useful information to improve and speed up risk analysis. BERT models, which are further refined by training on specific literature topics for greater comprehension, have been employed by Lee et al.,^15^ with improved results compared to the generic BERT models. Work by Huang and Cole^16^ was also found to improve data extraction for their research using a BERT model trained on battery literature, compared to the original model.

While many analyses of the cost of hydrogen use specific location data to compare costs at various points globally, this leads to a snapshot view of a given location and does not provide a complete picture of the global landscape for hydrogen production cost.^6,8^ Rogeau et al.^17^ created a map of the density of energy production around select areas of Europe, which illustrates the LCOH distribution in this area but leaves many areas unavailable for analysis. Devlin et al.^18^ used data from specific locations around the globe to generate a map of suitable areas for green hydrogen steel projects; however, there are large gaps in the map where no data are available.

To address the above gaps, this study presents a new framework for evaluating green hydrogen (i.e., hydrogen from water electrolysis) cost reduction potentials from both progressive and disruptive development pathways. This is achieved by a realistic model of the electrochemical systems, including the balance of plant units. AI models, including natural language processing (NLP) models, are used for high-quality and high-speed data extraction from large bodies of texts so that more accurate predictions of future cost trends are completed. Additionally, a global look at LCOH values was conducted to determine potential future regions for hydrogen production, as so far, there is little complete data available for this.

## 2 Methodology

### 2.1 Overall Framework

**Figure 1:**
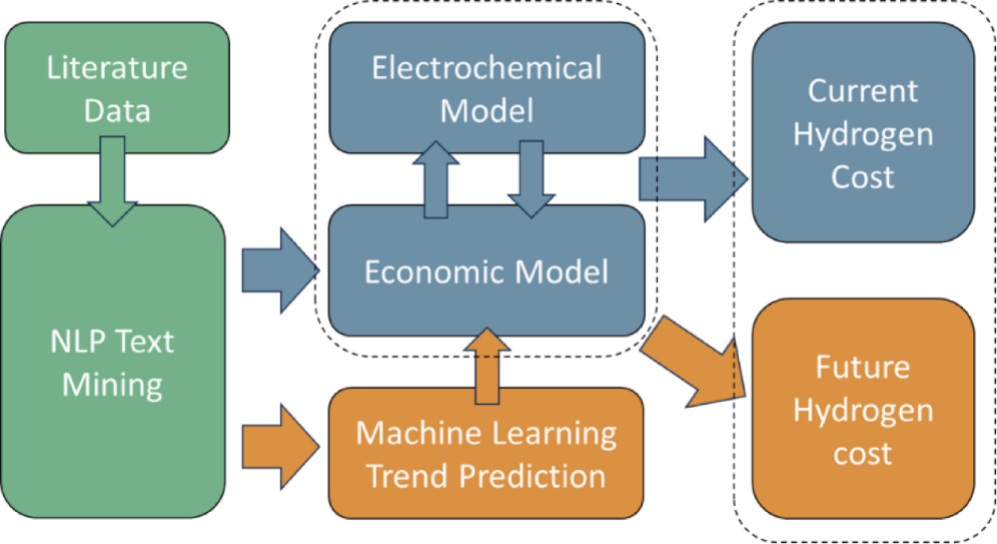
AI-assisted, data-driven framework for green hydrogen cost trend predictions.

Here, we propose a new AI-assisted framework for calculating the potential of green hydrogen cost reduction year on year, as shown in Figure 1, which illustrates the direction of flow for data and how the sperate models interact with each other to calculate the levelized costs of hydrogen (LCOH) over time. The starting point is the literature data, which forms the basis for all the information analyzed to calculate LCOH. This data are gathered via a BERT-based NLP model. The data will be passed directly through the economic and electrochemical models for LCOH calculation, as well as through probabilistic uncertainty models, so that both the baseline development scenario and probable regions of likely development may be calculated. The economic and electrochemical models use shared data to calculate related costs and mechanistic parameters, which are solved iteratively for the LCOH cost. Historic data are extracted from the literature by the NLP text mining tool and fitted using neural network predictions to create future trends for individual technological development. The overall LCOH trend is created using predictive data to calculate a series of technoeconomic values representing the change over time. This technique is repeated using randomly sampled inputs from the trend data to inform a Montecarlo simulation and create probabilistic regions of likely LCOH values.

### 2.2 Economic Model

The levelized cost of hydrogen (LCOH) is calculated using a combined economic and voltametric model that takes the physical parameters of proton exchange membrane (PEM) electrolyzers to generate operating information. The economic and voltametric model is defined in the equations below, derived from ref^12^.

The economic model is designed to calculate a net present value, NPV, using the yearly cash flow, adjusted for time in years (with a 20-year assumed lifetime), *t*, and a weighted average cost of capital (WACC) of 7.5%. This WACC indicates a moderate risk.^4^ The NPV is dependent on the sale price of hydrogen, which is adjusted to give an NPV value of 0. An NPV of 0 indicates the lowest price the hydrogen could be produced for, and the process could still be economically viable.
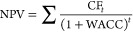
1

The cash flow, CF, is composed of the net earnings less the depreciation, both in $ year^–1^.
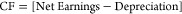
2

The cash flow includes year zero, where the total capital and working capital expenditures are treated as the yearly outgoings. Net earnings are the profit adjusted for tax by adding the previously removed depreciation, where the profit is the income less outgoings ($ day^–1^) multiplied by the working days per year, DPY, assumed to be 350.
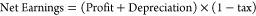
3where
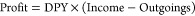
4

The depreciation is calculated using the straight-line method, whereby the total capital expenditure, CAPEX_tot_ ($), is divided by the lifetime of the plant, in this case, 20 years.
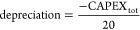
5

The income is calculated from the mass flow rate of hydrogen produced,  (kg day^–1^), minus losses, , and then multiplied by the hydrogen sale price per unit mass,  ($ kg^–1^).
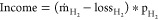
6

Outgoings represent the negative cash flow from expenses such as utilities and replacement cell parts. These expenses are collectively referred to as operating expenditure or OPEX_*i*_ ($ day^–1^), where *i* represents the electricity (elec), the membrane-electrode assembly replacement (MEA), the plates (plates), porous transport layer replacement (PTL), process water purchase (water), and maintenance and compressor costs (main and comp).
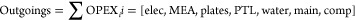
7

The capital expenditure, CAPEX_tot_ ($), is comprised of the capital cost of the cell parts, compressor, and total plant cost, cel, comp, and plant, all in $.
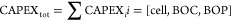
8

The full set of equations for the economic model can be referred to in the Supporting Information.

### 2.3 Electrochemical Model

The electrochemical model is used to simulate cell performance under various design and operation conditions, which is then fed into the economic model. The total cell voltage, *E*_cell_ (*V*), is defined as the sum of the thermodynamic, Δ*E*_thermo_, resistance, *E*_res_, mass transfer & Nernstian conversion (MTNC), *E*_MTNC_, and cathodic and anodic overpotentials, *η*_cathode_ & *η*_anode_, voltages, as shown in eq 9^12^.
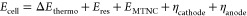
9

The *E*_mem_ is comprised of the membrane thickness multiplied by the current density, *J* (A m^–2^), and divided by the membrane conductivity, *σ*_mem_ (Ω^–1^ m^–1^), where λ is the humidification degree (−).
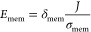
10
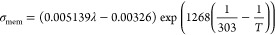
11

The MTNC potential is composed of the conversion and mass transfer, potentials, Δ*E*_conversion_ & Δ*E*_mass transfer_, and overpotentials, *η*_cathode, conversion_ & *η*_mass transfer_.
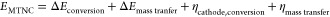
12

The conversion and mass transfer potentials are calculated as shown in eqs 13 & 14, using the molar densities of hydrogen, oxygen, and water, , all in (mol m^–3^). They represent the voltages required in order to activate the reaction and drive the mass transport of particles to the active surfaces of the catalyst materials.
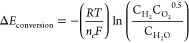
13
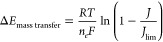
14

The limiting current density, *J*_lim_ (A m^–2^), represents the physical limitation of the reactant to reach the surface reaction sites, and hence, the theoretical maximum value the current density can reach. It is assumed from literature values to be high enough not to interfere with cell operation.^19−22^

The electrode overpotentials represent the deviation from the theoretical half-cell potential of the electrochemical reactions and are highly dependent on operating conditions and system configuration, as literature values for reference data vary widely.

The cathode conversion overpotential, *η*_cathode,conversion_, is calculated as eq 15 where *X*_*r*_ is the conversion of water to hydrogen (−), and TS_cathode_ is the Tafel slope for the cathode (V/dec). Eq 16 calculates the mass transfer overpotential, where *J* & *J*_lim_ are the current density and limiting current density, respectively.
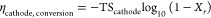
15
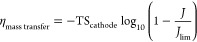
16

The cathode or anode over potential can be calculated from eq 17, where *η*_*i,*ref_ is the anode or cathode reference over potential taken from the literature, given at a reference current density *J*_*i,*ref_ (A m^–2^), *η*_*i,*kin_ is the anode or cathode kinetic overpotential.
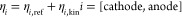
17
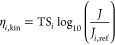
18

The full set of equations for the electrochemical model can be referred to in the Supporting Information.

### 2.4 AI Data Collection and Trend Prediction

In order to determine the trends of future technological development, historic data of reported electrolyzer performance was gathered from the literature using a Bidirectional encoder representations from transformers (BERT)-based natural language program (NLP) model. The areas of electrolyzer technology for which data have been collected are operating current density and precious metal catalyst loading for both iridium and platinum. 600 literature articles on the topic of water electrolysis found using the search terms “PEM Water Electrolysis” between 2012 and 2023 were passed through the model sequentially using Python packages developed by Huang and Cole.^16^ The use of machine learning to generate data automatically allowed 1000 s of data points to be generated from the corpus of papers in approximately 4 h.

Preprocessing included removing corrupted or duplicate files and replacing units, such as mg_Ir_ cm^–2^ or g/m^2^, with an associated alternative, such as “unita” or “unitb”, etc., as the model had difficulty parsing the formatting associated with subscripts and superscripts. These replacements could then be reversed to ensure dimensional consistency in the results. Overall, there were 14 substitutions for catalyst loading units and 4 substitutions for current density units. The model searched each sentence for information relating to the key terms “Catalyst Loading,” “Iridium,” “Platinum,” and “Current Density,” and REGEX search strings were developed to allow a wide range of related terms to be found (such as plurals, abbreviations, etc.). The NLP model returns data in a structured format to allow easy extraction of the key points, as long as the sentence meets a minimum confidence threshold for matching the search terms.

**Figure 2:**
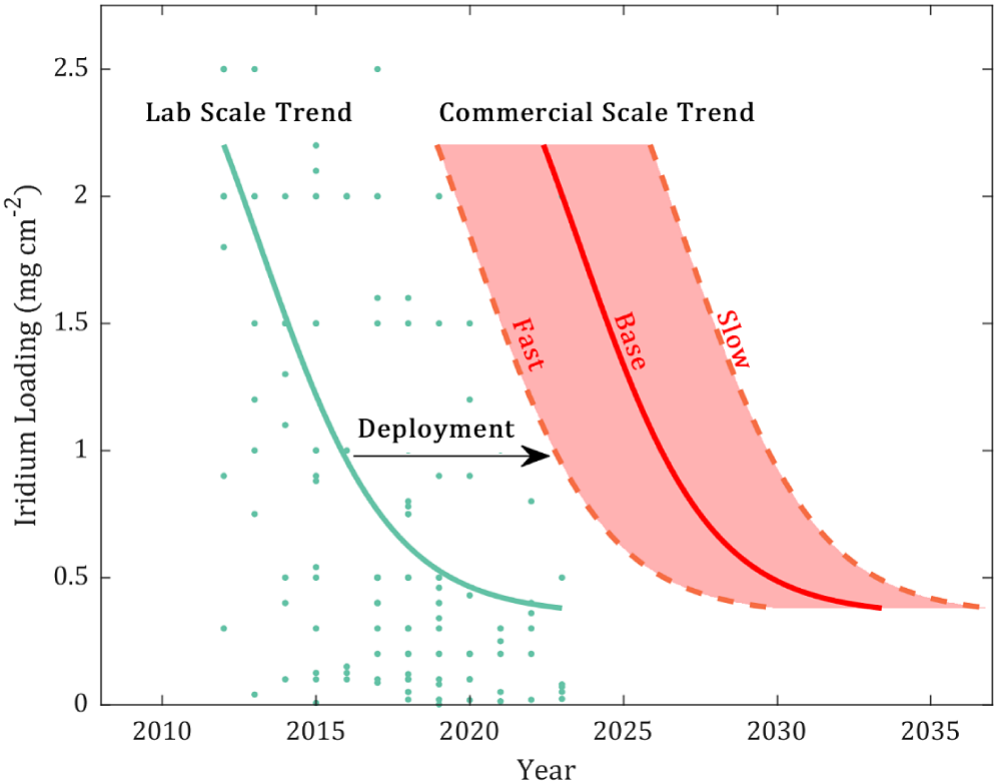
Lab-scale data and trend fitting of iridium catalyst loading values found in the literature with a deployment gap to a commercial scale scenario.

This data collection technique allowed the creation of bespoke databases of information about historic catalyst loadings and current densities in PEM electrolyzers. These data were fitted with trend curves using neural networks generated from the MATLAB regression learner toolbox to ensure that the determined trends were physically valid. In order to generate prediction intervals, jackknife resampling is undertaken, where an individual data point is removed and replaced with a data point randomly generated within the maximum and minima for the location of the removed point. The curve is then regenerated for the new data and creates a different fitting, which is repeated 10000 times, at which point all the generated curves are collected to create an area of likely future values and used to generate Figure 2 for iridium loading and Figure S3 for more examples of other parameters. The fitted curves for platinum did not show a power law relationship, which is attributed to the loading of platinum not typically being the subject of optimization due to its lower loading and cost than iridium.

The predicted values of catalyst loading and current densities are taken from academic papers, which represent the best performance achieved in lab-scale research. It should be noted that there is a gap between lab results and commercially available performance due to the time taken to deploy a technology at commercial scales, as shown in Figure 2. To reflect such a gap, the curves for commercially available performances were derived by shifting the curves of lab-scale results over time.^19^ Here we consider a ten-year gap between the lab scale targets and the commercially viable technology for current density and iridium loading and 5 years for platinum loading, which is based on the gap between current commercially available values and the predicted value from literature and also aligns with other studies.^19^ In addition, we consider fast and slow uptake scenarios with a ±50% rate of uptake compared to the base-case deployment to account for scenarios where the transition from lab-scale state-of-the art to commercially available products is accelerated due to significant focus or occurs more slowly due to technological issues that are hard to overcome. Neural network trend fitting was applied to the NLP-collected data due to the high level of noise present, whereas the membrane price and the levelized cost of electricity (LCOE) data used simpler regression fitting for trend prediction. Due to the difference in fitting methods, the LCOE and membrane have 95% confidence intervals, which were not possible for the NLP-collected data. The base case LCOE value used is the 2021 global average onshore wind energy LCOE^23^ of approximately $0.03 kWh^–1^.

## 3 Results and Discussion

### 3.1 Progressive Innovation-Driven Cost Reduction

The base scenario inputs are summarized in Table S1. The LCOH is calculated for an electrolyzer system at a range of scales from 1 kW to 10 MW to determine the point at which the system becomes independent of scale, which can be seen in Figure S1. After 2 MW, further scale increases resulted in small LCOH decreases, and this value was considered suitable for all analyses herein.

**Figure 3:**
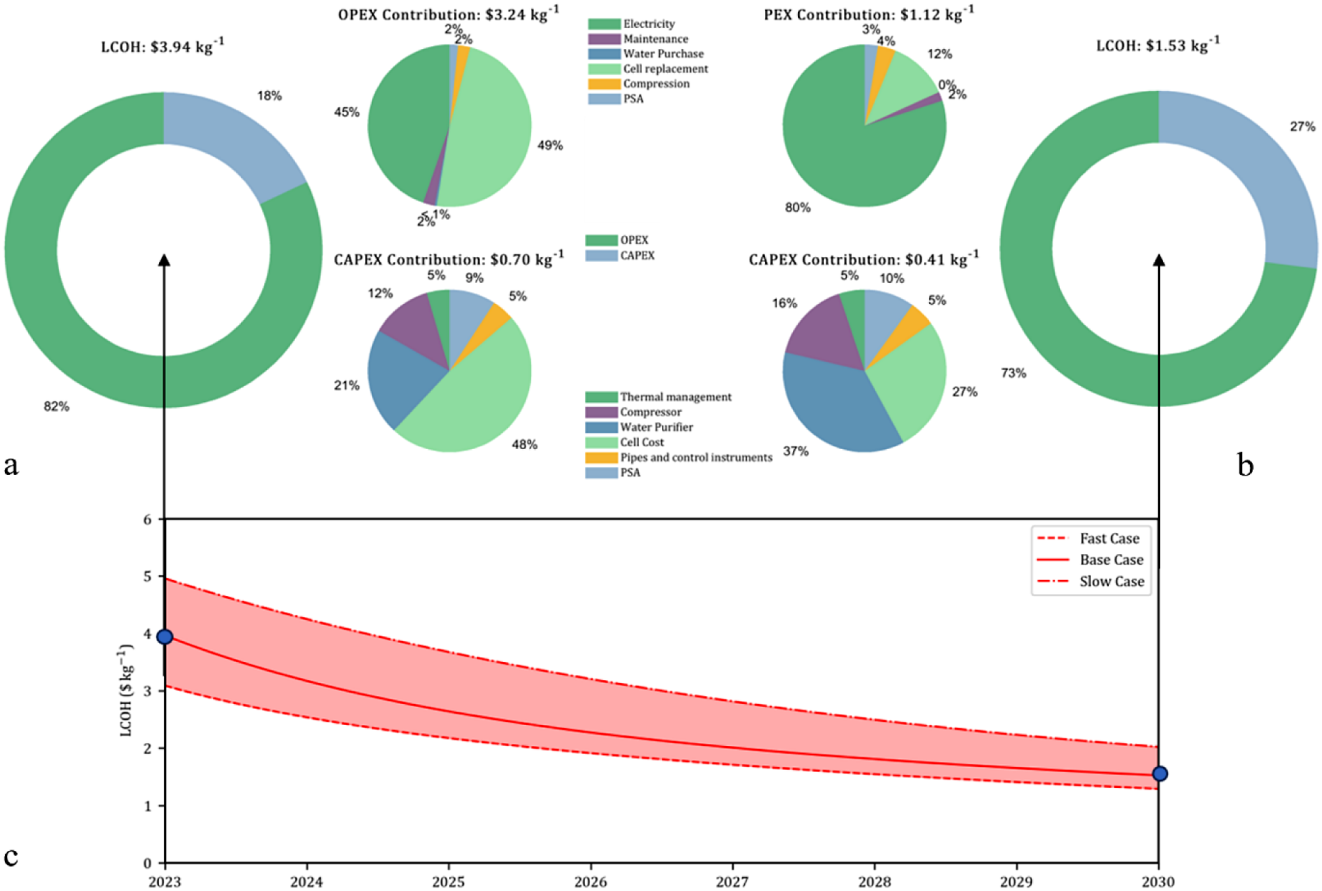
LCOH cost breakdown into OPEX, CAPEX, and their respective components for a) 2023, b) 2030, and c) LCOH reduction with fast, base case, and slow scenario pathways between 2023 and 2030.

Figure 3a shows that the 2023 LCOH has a value of $3.94 kg^–1^ and is composed of the capital and operating expenditures (CAPEX and OPEX), which occupy 18 and 82% of the total LCOH, respectively, in line with current literature estimates.^4^ 2030 LCOH is predicted to be significantly lower, as shown in Figure 3b. The capital expenditure represents the purchase cost of the electrolyzer and balance of plant (BOP) units. Most of the BOP units’ costs are nonlinearly dependent on the mass of hydrogen processed, which means that as the electrolyzer system size increases, the BOP makes up less of the total cost. The operating expenditure (OPEX) is responsible for the majority of the LCOH cost and consists of the cell running costs.

Figure 3c displays the LCOH change over time based on the predicted technological deployments, with fast and slow uptake scenarios representing a ± 50% change in the rate of technological deployment. Figure S8 displays reference data,^4−6,8,9,17,24^ for predicted and targeted LCOH changes, which show a wide range of current and 2030 LCOH values, in comparison to the deployment cases in Figure 3b. While not able to fully supplant the cost of gray hydrogen by reaching the $1 kg^–1^ earth shot target, the scenarios do show a promising trend toward significantly less costly green hydrogen by 2030. The deployment rate is crucial to the speed at which the LCOH initially decreases. However, it does not significantly affect the final LCOH as each scenario approaches the same plateau in value, which is the limitation of the progressive improvements.

As can be seen in Figure 3c, the LCOH values start at approximately $4 kg^–1^, and by 2030, the LCOH is predicted to fall below the $2.5 kg^–1^ price mark, which allows it to be competitive with gray hydrogen.^4^ Compared to the majority of LCOH data reported in the literature, our prediction has a more moderate current LCOH value that falls to a competitive value price range by 2030, but still needs a significant improvement to reach the $1 kg^–1^ earth shot target.^4,5^ These differences are due to the details considered when calculating the LCOH, which result in the accurate inclusion of a wide range of costs.

Cell material replacement and electrical costs contribute over $3 to the LCOH, the overwhelming majority of the cost. Cell material costs are reduced through improving their lifetime and reducing the quantity of material required for the same cell performance; this is particularly true of catalyst material. Electricity costs can be lowered via voltage reduction or current density increases, both of which amount to an increase in production per unit energy expended. The levelized cost of electricity (LCOE) is also pivotally important here, with LCOE alone contributing approximately $1.46 to the LCOH.^25^ The global average weighted LCOE of onshore wind has fallen steadily over time, as shown in Figure S4e, as has the LCOE of all renewables.^23^ If the trend of LCOE falling continues over the coming years, the LCOH would be dramatically lowered.

The balance of plant capital and operating costs also contributes significantly to the LCOH, making up the majority of the remainder of the LCOH in 2030. These units are mature technologies, such as compressors, which are unlikely to materially improve in cost compared to the electrolyzer. However, their necessity may be reduced or removed as the electrolyzer technology improves, and so their contribution to LCOH decreases. These and similar properties’ future values can be predicted to determine how the LCOH will change over time, as shown in Figure S4f. The rate of technological deployment, which results in technological changes and hence LCOH changes, will be key to determining the cost of hydrogen in the future.

The LCOH will change over time, as a function of the changes of the inputs’ changes over time, as shown in Figure S4. Recent work on high-current-density electrolyzers has shown electrolyzers capable of reaching extremely high current densities^26,27^ of 15–20 A cm^–2^. It has also been shown that gas permeation increases to high levels, with a current density at only 4–6 A cm^–2^, more so in thinner membranes, and that thinner membranes reduce cell voltage.^28^ This shows that while it is possible to move from current densities of 1 A cm^2^ to high current densities in excess of 10 A cm^–2^, further development is required to do so in a safe manner that does not permit explosive gas mixtures to form.

Catalyst materials offer two pathways for cost reduction: first, a reduction in the price of the material itself, which in the case of platinum group metals (PGM) is high, and second, a reduction in the required amount of materials to achieve the cell performance. Catalyst loading has shown a trend of falling steadily as electrolyzer technology has developed over time (Figure S4a & b) and as lower catalyst loadings are able to provide the required lifetime and performance. While the price of PGMs is expected to fluctuate, cheaper alternative metals for catalysts, such as nickel and cobalt are being explored, but the difficulty of developing non-PGM catalysta for PEM electrolysis is expected to be high.^4,29−31^ Current iridium catalyst production techniques result in loadings^32^ in the region of 1–2 mg cm^–2^. Different catalyst production techniques are able to produce significantly lower loadings (<0.25 mg cm^–2^), with comparable or improved cell performance.^26,32^ Recent research into ultralow iridium loading has developed cells with catalyst loadings several orders of magnitude lower, as low as 1 μg cm^–2^ but more typically around 10–100s μg cm^–2^, to ensure stability.^32−35^

The other main cell component costs are the membrane, the porous transport layer (PTL), and the flow plates. PEM electrolyzers typically use Nafion membranes, which are widely used in fuel cell applications.^36^ The price of these types of membranes is not expected to decrease significantly with time; however, as order volume increases, the price per unit membrane is expected to decrease, as shown in Figure S4d.^37^ While Nafion membranes have high performance characteristics and durability, they are expensive, and alternative technologies are being actively investigated.^38^ Nonfluorinated aromatic polymers offer promise but are unable to compete on all fronts with Nafion membranes.^39^ Work by Ko et al.^40^ and Choi et al.^41^ demonstrates the promise of low-cost and high-performance alternatives to Nafion membranes; however, it also shows they are not currently viable due to limited lifespans.

PTLs are used to disperse the fluid flow evenly from the flow plates across the catalyst as well as facilitate the movement of fluids in and out of the electrolyzer, and as a consequence, both the PTLs and plates are made using titanium to enhance durability in the acidic environment of PEM electrolyzers.^42,43^ As a material, titanium is less expensive than PGMs but is difficult to machine, so it incurs a higher cost than other metals.^44^ These factors contribute to a significant cost in the cell, which could be reduced if the parts were replaced with coated stainless steel.^45,46^ Stiber et al.^46^ conducted work on stainless steel electrolyzer components, which had comparable performance to their titanium alternatives but lower lifetimes.

### 3.2 Disruptive Innovation-Driven and Long-Term Cost Reduction

**Figure 4:**
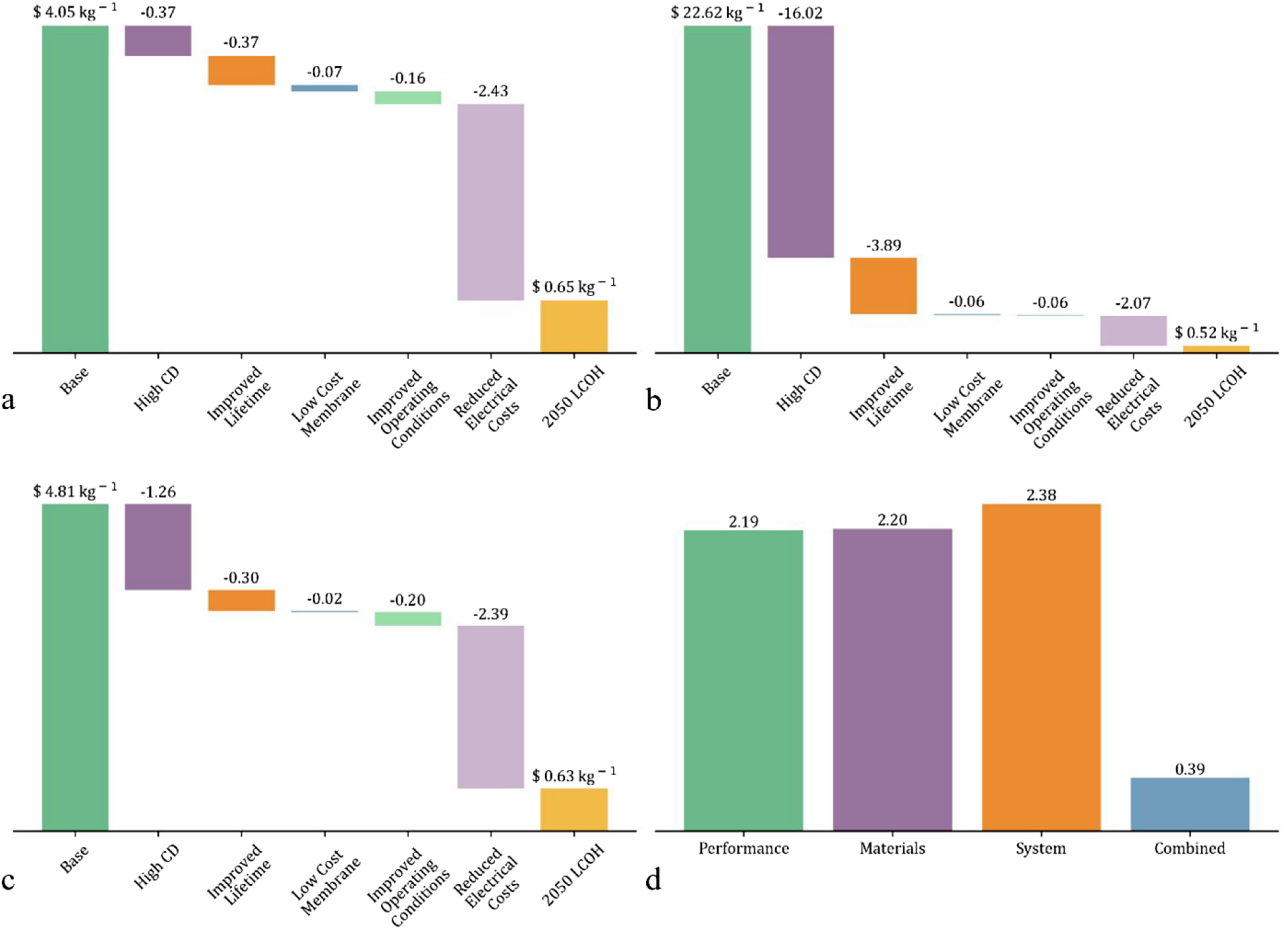
LCOH reduction pathways through improved technological development toward 2050 for a) AEM, b) seawater, c) BPM electrolyzers, and d) potential groupings of technological improvements compared to the base case of LCOH.

For a large change in LCOH in a shorter period of time, a disruptive technology could be developed that allows a significant step change in LCOH. Technologies that can supplant the performance of currently available high-quality electrolyzers are being researched but are at low technological maturity levels. Technologies, such as anion exchange membrane (AEM) electrolyzers, could offer an electrolysis route, which is less harsh than the acidic conditions of PEM electrolyzers, thus improving cell lifetime and potentially reducing catalyst cost. Seawater electrolysis would reduce the balance of plant and operating costs, and bipolar membrane (BPM) electrolysis could potentially combine the benefits of PEM & AEM technologies. If a breakthrough can be achieved for one of these technologies, then a step change in LCOH could become possible, as shown in Figure 4. The individual technological changes required for the LCOH to radically drop below its predicted 2030 level are considered complex challenges for a PEM cell and summarized in Table 1.^4^ These technological changes represent the targets for technological development by 2050 for the base case and AEM electrolyzers, according to IRENA, with the seawater and BPM targets extrapolated from these values.^4^

**Table 1:** Current Operating Assumptions and 2050 Targets for the Base Case (PEM Systems) and Disruptive Technological Alternatives

	base case		AEM		seawater		BPM	
	2023	2050	2023	2050	2023	2050	2023	2050
current density (A cm^–2^)	1	5	1	2	0.3	1.5	1	5
lifetime (1000 hours)	40	100	10	100	1	60	10	100
membrane cost ($ m^–2^)	1000	500	2000	100	1000	100	2000	500
operating pressure (bar)	30	70	30	70	30	70	30	70
operating temperature (°C)	80	80	60	80	60	80	60	80

Figure 4a shows the LCOH reduction pathway by technological improvements for AEM electrolyzers possible by 2050, as shown in Table 1. By employing alkaline conditions, anion exchange membrane (AEM) electrolyzers are less corrosive and able to be composed of cheaper materials, which may result in a lower LCOH as these costs are responsible for large portions of PEM hydrogen’s LCOH.^47^ As an immature technology (TRL 2/3 vs 7/8 for PEM), assumptions for the cost of these materials have to be made, as current AEM materials cost is higher than PEM, but this would not be the case if production were to reach comparable scales in the future.^48,49^ As shown in Table 1, the AEM is assumed to operate at an equivalent current density to the PEM, but due to the use of hydroxide ions as the charge carrier, it has less favorable reaction kinetics, which results in slightly worse cell performance.^50,51^ The AEM assumptions include stainless steel PTL, non-PGM catalyst layers, and durable anion membranes, which can offer comparable performance and lifetime to state-of-the-art versions, as these technologies are considered possible in the future for AEM.^52^ The AEM LCOH is slightly higher than the base case PEM, at $4.05 kg^–1^, and the largest effect on LCOH would be through reducing electrical costs, leading to a potential LCOH below $1 kg^–1^ in 2050.

The LCOH reduction pathway by seawater electrolyzers can be seen in Figure 4b. Seawater electrolyzers directly electrolyze seawater (assumed to be freely available) to produce hydrogen; therefore, removing the need for water purification, which is expensive both in terms of energy and capital cost. The initial seawater electrolyzer LCOH is extremely high, > $20 kg^–1^, which is attributable to the low current density and durability of components that the technology is currently able to achieve.^53^ As recent literature has shown significant improvement in both lifetime and cell performance, it is anticipated that these challenges can be overcome with greater research, and the performance of seawater electrolyzers will reach comparable levels to the AEM water electrolyzer targets, potentially leading to an LCOH below the predicted LCOH for PEM electrolyzers by 2050.^51^

BPM electrolyzers combine an acidic PEM-like anode side and alkaline AEM-like cathode side, with water fed directly to the membrane for splitting, and attempt to overcome the slow charge transport of AEM and the harsh conditions of PEM by combining the strengths of both technologies.^6^ At $4.81 kg^–1^, the LCOH can be decreased greatly through current density and electrical cost improvements. BPM electrolyzers can achieve high current densities in excess of 9 A cm^–2^, but still require expensive precious metal materials on the acidic side and have slow OH^–^ transport to the cathode side, ultimately failing to overcome the weaknesses of AEM and PEM technologies.^54^Figure 4c shows a significantly reduced LCOH for BPM electrolyzers by 2050 if targeted development can be achieved.

Furthermore, the effect of potential groupings of the disruptive innovations and step changes on the hydrogen cost were considered, as displayed in Figure 4d, with each scenario able to reduce the LCOH by $2.75, 2.74, and 2.56 kg^–1^, respectively. This figure shows that if combinations of disruptor changes occur, extreme lower LCOH values can be found.

The performance improvements use the IRENA 2050 electrolyzer targets of 1.7 V and 5 A cm^–2^, whereas the material improvements are based on replacing PGMs and titanium components with non-PGM catalysts and stainless steel parts. The system improvements are taken as ways of reducing BOP costs, such as using seawater instead of highly purified water, operating at 70 bar to reduce compression costs, improved membranes, which eliminate gas crossover to negate the need for gas purification, and LCOE reaching an extremely low level. Large improvements can be achieved through material improvements, as the catalyst and titanium material costs are extremely high. Catalyst price change could occur either through the disruptive route of new non-PGM material use or through the progressive development of lower material loading. While non-PGM catalysts would be extremely beneficial to the cost of the cell materials, it is considered an extremely hard goal to achieve and is unlikely to occur in the near future.^4^ The current density and voltage reductions also show a potential avenue for large LCOH reductions, which could be achieved either through a new technology being developed that operates easily at high current densities, such as bipolar membrane electrolyzers.^55^ The combined effect of all changes being achieved shows an extremely low value of $0.39 kg^–1^, which provides an idealized scenario of what is possible if all technological improvements can be made.

### 3.3 Market Uncertainty Analysis

**Figure 5:**
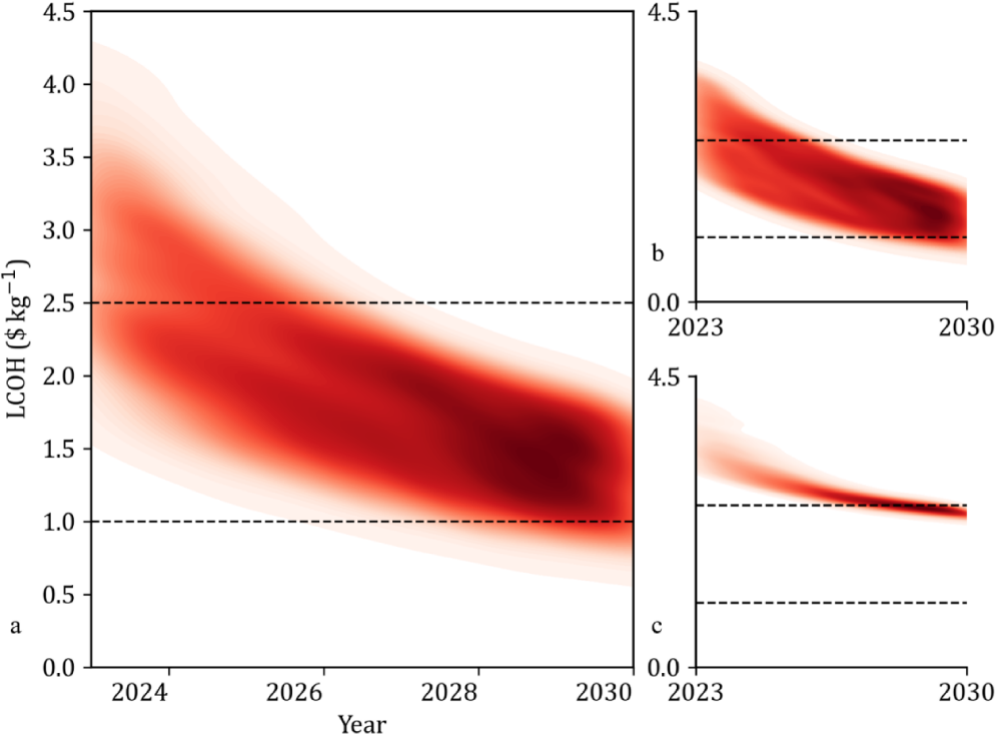
a) Combined b) electricity and c) precious metal uncertainty heatmaps.

Within the factors that are used to calculate the LCOH is an inherent amount of uncertainty due to variations in energy and material costs, which result in hard to predict electricity and precious metal prices. To analyze this market uncertainty, the cost of precious metals and electricity used to predict their future prices is analyzed for its variance and used to predict the potential region prices could vary into over time. Figure 5a shows the uncertainty inherent in making assumptions for changes over time and represents the combined market uncertainty in precious metal and electricity prices. Initially, there is a very widespread of LCOH-feasible regions, with a diffuse center around $2–4 kg^–1^.

This diffuse region begins to consolidate between 2025 and 2026 with a higher probability region and shows that by 2030, lower LCOH costs are highly likely. Typically, predictions of future cost intervals would be expected to start with a narrow variance that grows over time; however, this behavior is not displayed here for several reasons, which can be more easily described by separating out the causes of uncertainty, as seen in Figure 5b & Figure 5c for electricity costs and precious metals, respectively.

The price of electricity has a huge effect on the LCOH, and in Figure 5b, the LCOH has an extremely wide region of possible values, which are initially more concentrated before the trend diverges slightly over time. The spreading LCOH probability results in a more evenly distributed area toward 2030. Here, the prediction interval increases from 2023 to 2030, resulting in the spreading of LCOH value probabilities. However, due to the electricity cost already being at an extremely low level, the limits are unable to increase further as the predicted value approaches zero. This causes the uncertainty region in Figure 5b to not diffuse strongly over time but brings confidence to the prediction that the LCOH will fall in the sub-$2.5 kg^–1^ price region and become competitive with traditionally produced gray hydrogen.

In Figure 5c, the electricity price is kept constant to show the effect of uncertainty in the price of metals. The LCOH heatmap is initially very diffuse as the uncertainty in the price of precious metals causes large variation in LCOH, where lower material costs cause a noticeably larger change in LCOH than higher material costs. However, in the future, as the catalyst loading decreases, the proportional effect of the precious metal cost on the LCOH decreases, so despite the variation in metal cost increasing, the overall effect is a variance decrease, which results in convergence.

In order to minimize the effect of these uncertainties on LCOH, stable suppliers of precious metals and renewable energy should be sought, and recycling of materials should be considered to reduce material losses.

### 3.4 Geographic Analysis

**Figure 6:**
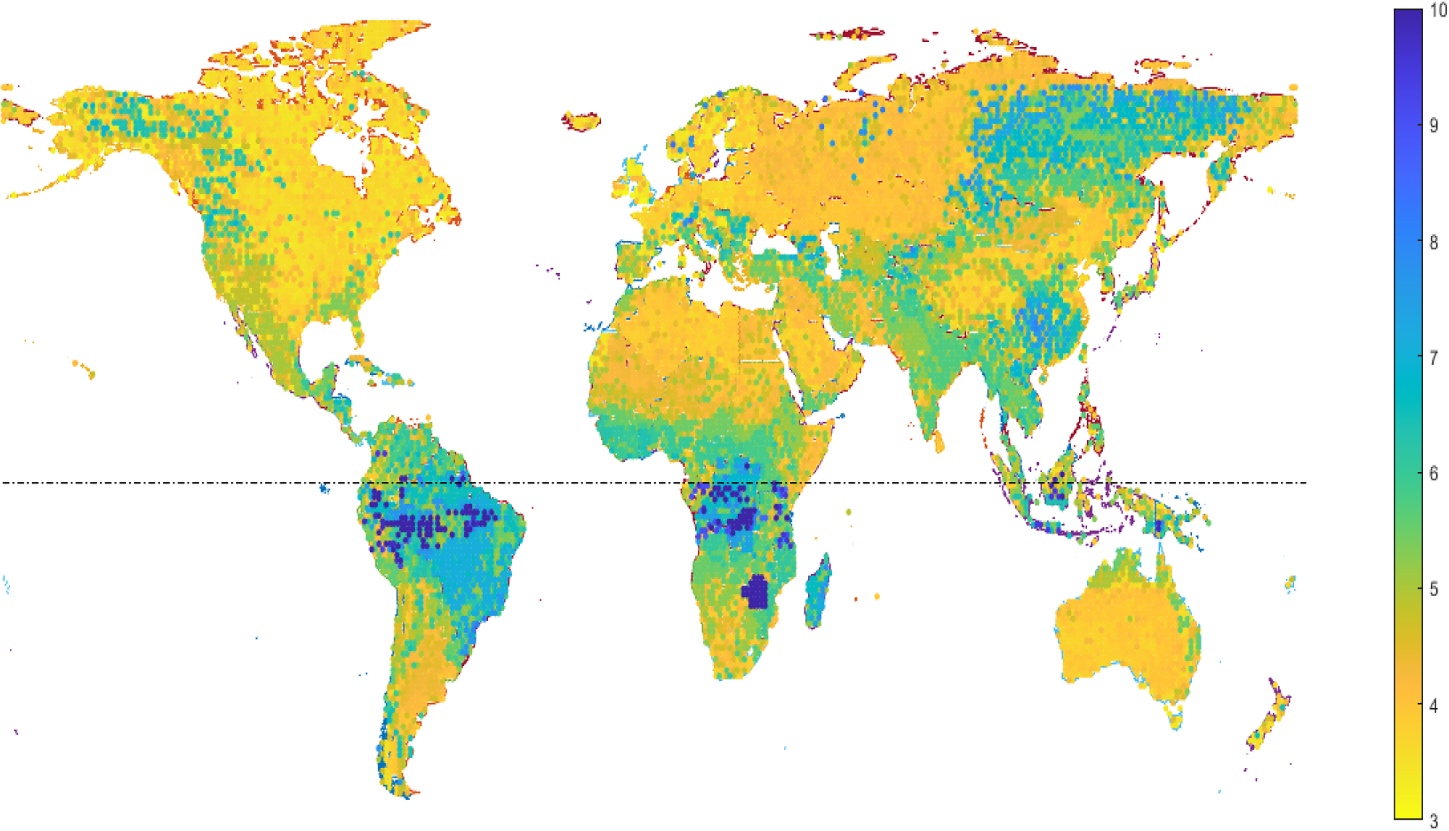
Global LCOH ($ kg^–1^) distribution in 2023 (equator at dashed line).

In order to map the cost of generating hydrogen globally, the LCOH based on solar and wind energy was determined for the global map, as shown in Figure 6. Figure 6 was generated using the MATLAB mapping toolbox. The LCOHs were calculated using the base case assumptions for electrolyzer type and performance for 2023. The discount rate chosen for the NPV analysis in this paper is the weighted average cost of capital (WACC), which varies by country. Solar and wind energy are location dependent and highly variable within a country, however WACC is constant for a given country.^56−58^ The solar and wind energy values and consequent LCOE values were determined using the EU PVGIS database and the world bank wind speed map.^59,60^ WACC values for each country were determined from Ondraczek et al.,^56^ IEA,^9^ and the International Renewable Energy Agency.^4^

By mapping the variance of LCOE and WACC across the globe, we can determine the cost for locations to produce hydrogen. It should be noted that the LCOE for solar energy is typically higher than for onshore wind energy, and the lowest solar energy LCOE found was 33% higher than the onshore wind energy LCOE used throughout the rest of this work.^9,23^ The more favorable latitudes are those in arid areas such as North America, North Africa, parts of southern Africa, parts of south and eastern Asia, Australia, and parts of South America.

These locations see considerable amounts of uninterrupted sunshine year-round, have higher wind average speeds, and have the lowest LCOEs and consequently lower LCOHs. Most of Europe and countries with high latitudes have fewer sunlight hours but more favorable wind speed conditions, and some economies with lower WACCs are able to generate low LCOH values in these locations. The inverse is also true; some countries in arid areas have unfavorable WACC values, which result in high LCOH values. The tropical areas around the equator have slightly lower sunlight availability than the arid areas further north and south due to the axial tilt of the earth and poorer LCOH values, despite some locations with favorable WACC values. As Figure 6 shows, the lowest value of ∼$3.2 kg^–1^ can be found in Ireland, but similarly low values can also be found in North America, Africa, and Asia.

The ability to model the LCOH’s spatial distribution is key to understanding the regional variation, to understand both the physical distrbution of renewable energy, and the economics of renewable investment in different countries.

## 4 Conclusions

This study presented a novel AI-assisted LCOH prediction to determine the cost of hydrogen both now and in 2030, as well as geospatially. The Initial LCOH at approximately $4 kg^–1^ falls in line with current literature estimates of LCOH. Analysis of the LCOH allowed the areas with the most potential for cost reduction to be identified, and the AI-assisted prediction of the LCOH from 2023 to 2030 allowed a prediction of how the LCOH will fall in the coming years for several different development scenarios. Even with the most pessimistic development scenario, by 2030, the LCOH of green hydrogen would likely still be competitive with gray hydrogen at the sub- $2.5 kg^–1^ level. The NLP model is currently a manual process, but through the use of an automated web scraper, the model could become semiautomated and updated regularly. There are several promising but immature technologies that could each represent a step change in the cost development of electrolyzer technology, AEM, BPM, and seawater electrolyzers, as long as technological targets are met by 2050. Each of the technologies represents opportunities for alternatives to PEM electrolyzers, which may be better suited for deployment in different situations. The analysis of the uncertainty shows that the fluctuation of the precious metal market and renewable LCOE could drastically affect the LCOH but are unlikely to cause the price to rise to a point where it becomes uncompetitive with gray hydrogen by 2030. The geographic analysis allowed the global distribution of LCOH to be determined, with the lowest-cost regions highlighted as potential areas to be investigated for electrolysis deployment, to allow better-focused future investment.

## Supporting Information Available

The full set of equations for the electrochemical model (PDF)
