# Supplementary material for: Potential of Progressive and Disruptive Innovation-Driven Cost Reductions of Green Hydrogen Production
Source: Energy Fuels. 2024 May 18;38(11):10370–80. doi: 10.1021/acs.energyfuels.4c01247 (PMC11163429; doi:10.1021/acs.energyfuels.4c01247)
Supplement: Supplementary file 1 — ef4c01247_si_001.pdf [file ef4c01247_si_001.pdf]

Potentials of progressive and disruptive innovation-driven cost reductions of green hydrogen production

(Supplementary Information)

Thorin Daniel [a], Lei Xing [a], Qiong Cai [a], Lirong Liu [b], Jin Xuan [,a]*

[a] School of Chemistry and Chemical Engineering, University of Surrey, Guildford, UK, GU2 7XH

[b] Centre for Environment and Sustainability, University of Surrey, Guildford, UK, GU2 7XH

*corresponding author, j.xuan@surrey.ac.uk

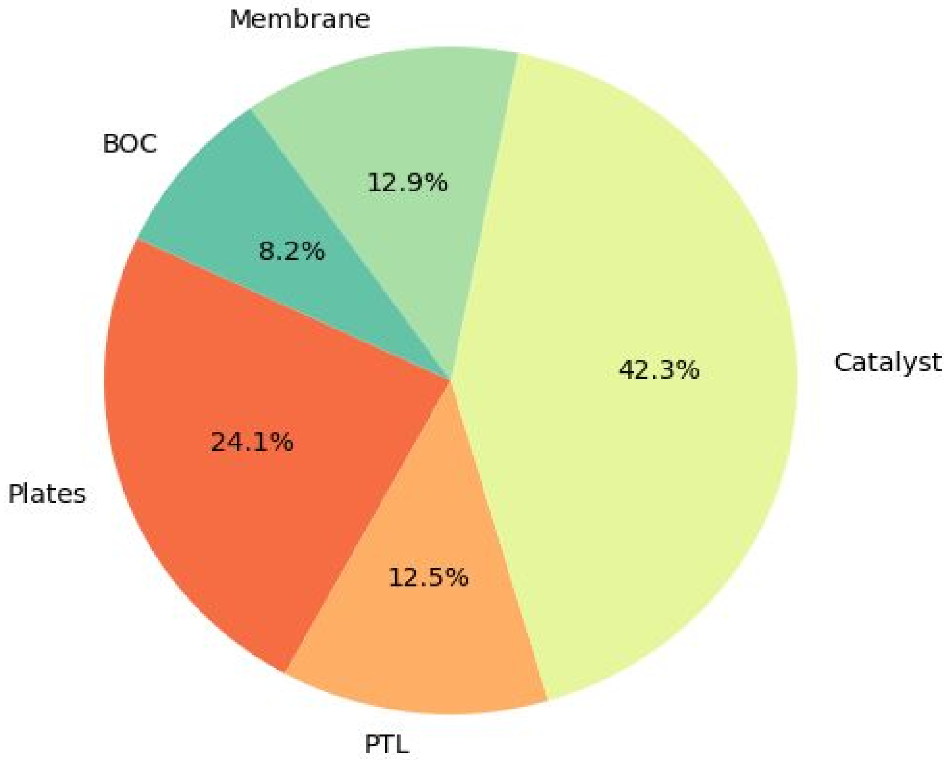

Figure S1: Balance of cell costs for 2 MW PEM electrolyser.

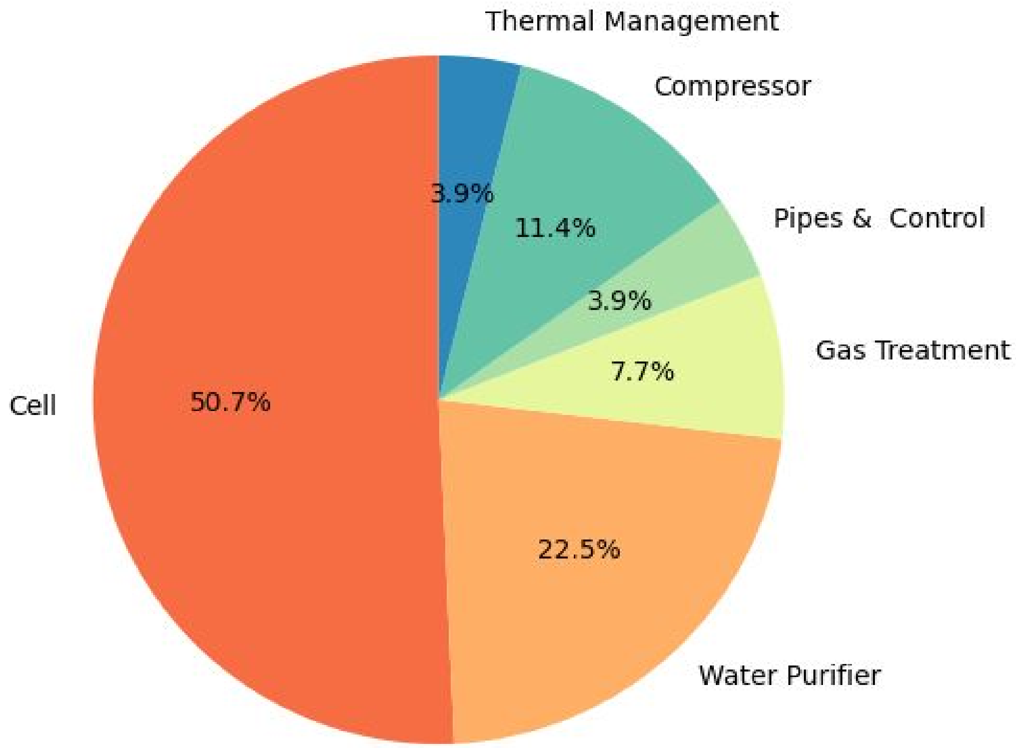

Figure S2: Balance of plant costs for 2 MW PEM electrolyser.

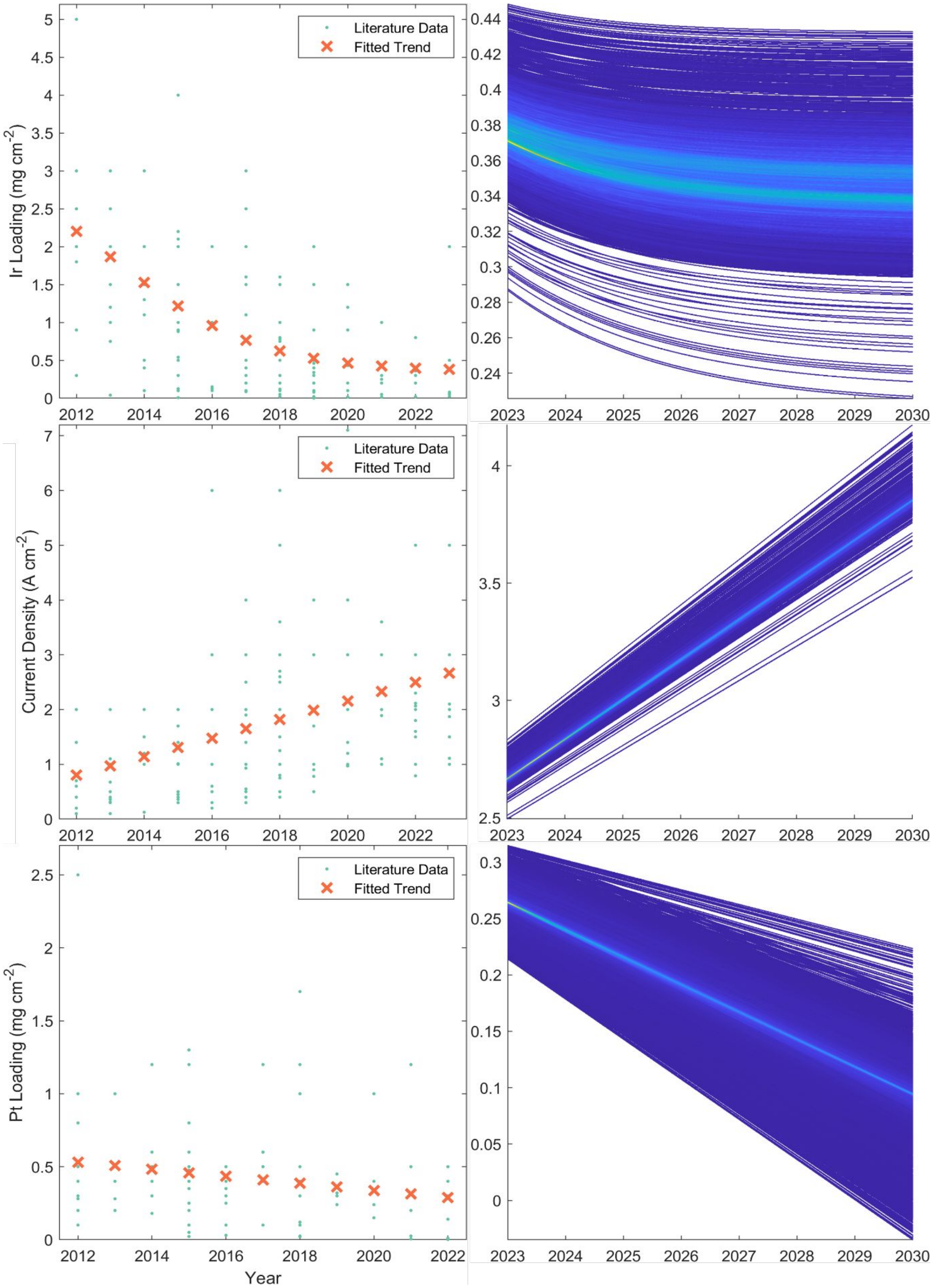

Figure S3: Historical literature data and resampling prediction interval.

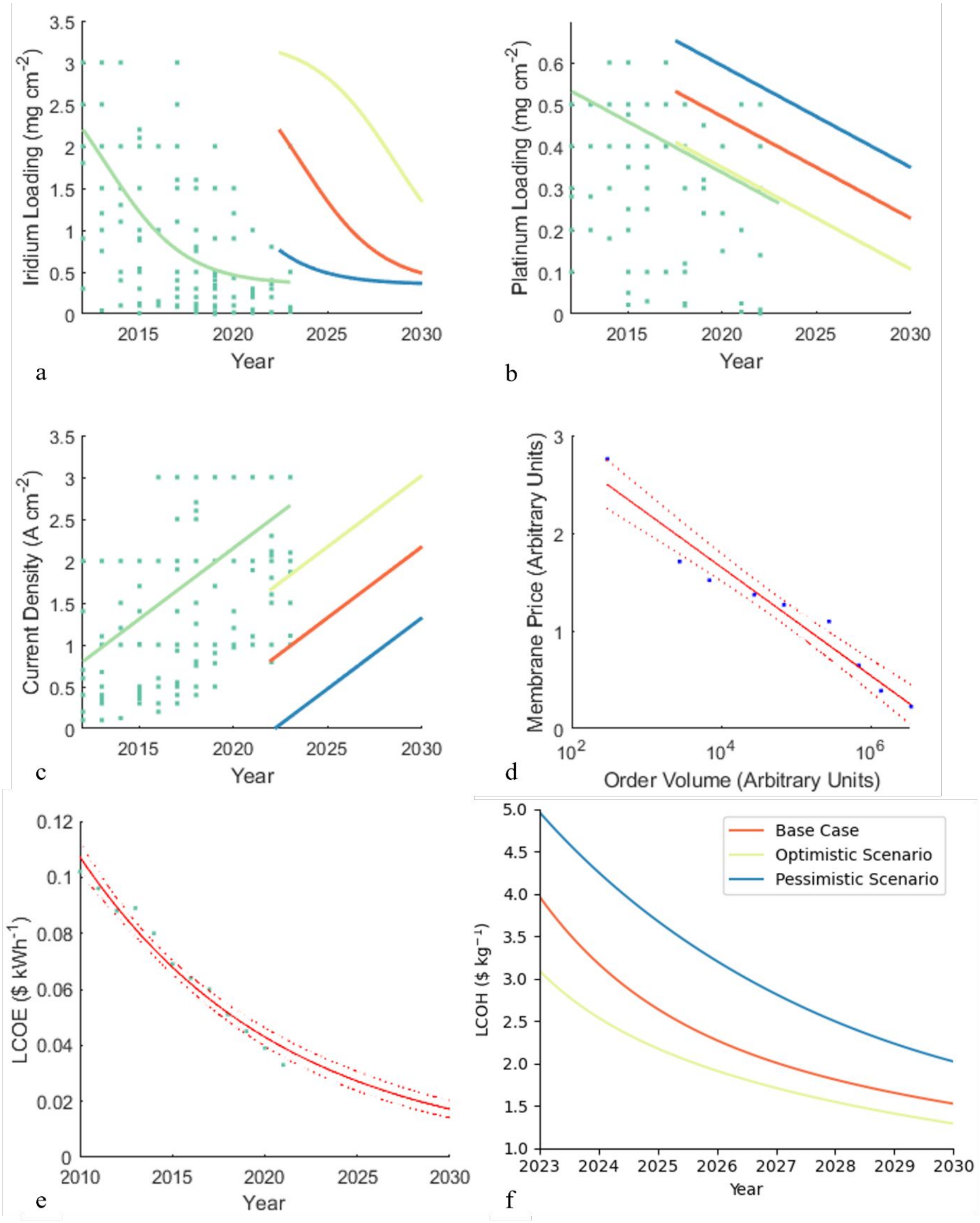

Figure S4: Historical data, fitted learning curve, offset deployment curve with optimistic and pessimistic scenarios for a) Iridium loading b) Platinum loading c) Current Density and d) Relative membrane cost for order volume with 95% confidence interval e) global weighted LCOE with 95% confidence interval f) LCOH trends for base, optimistic, and pessimistic scenarios.

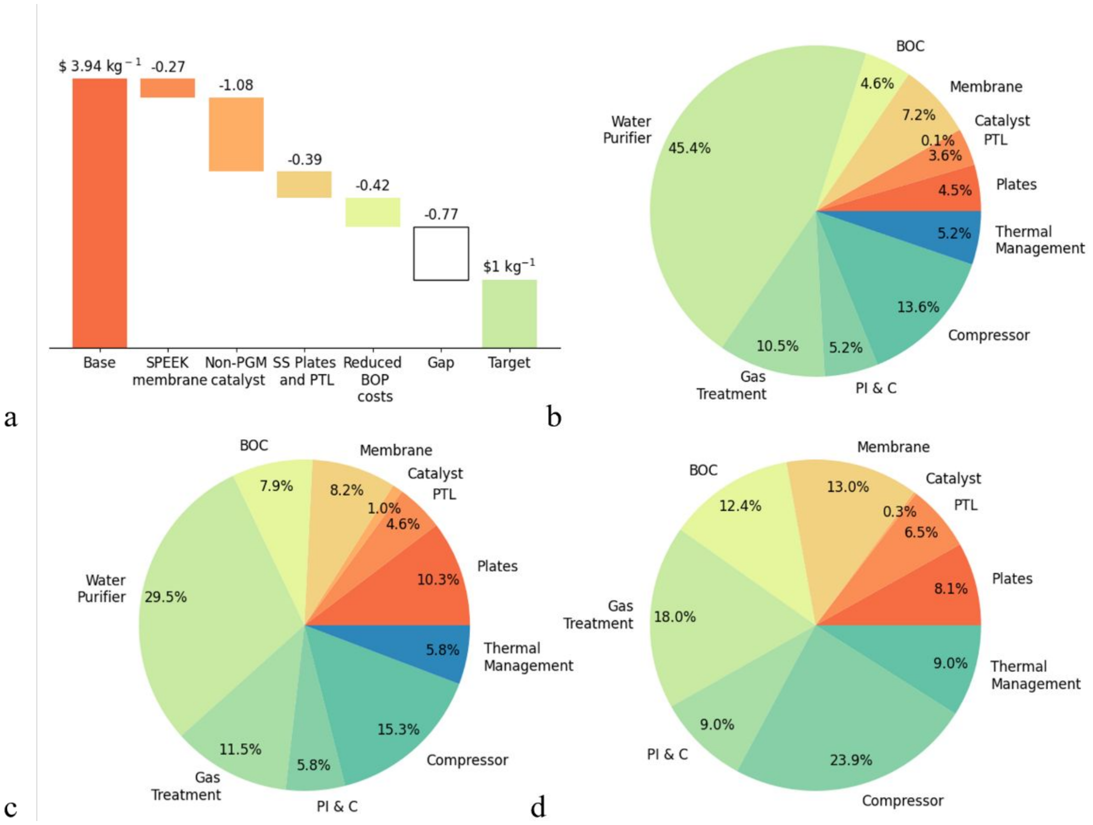

Figure S5: a) PEM Gap Analysis and Scenario breakdown for b) AEM, c) BPM, and d) seawater electrolysers.

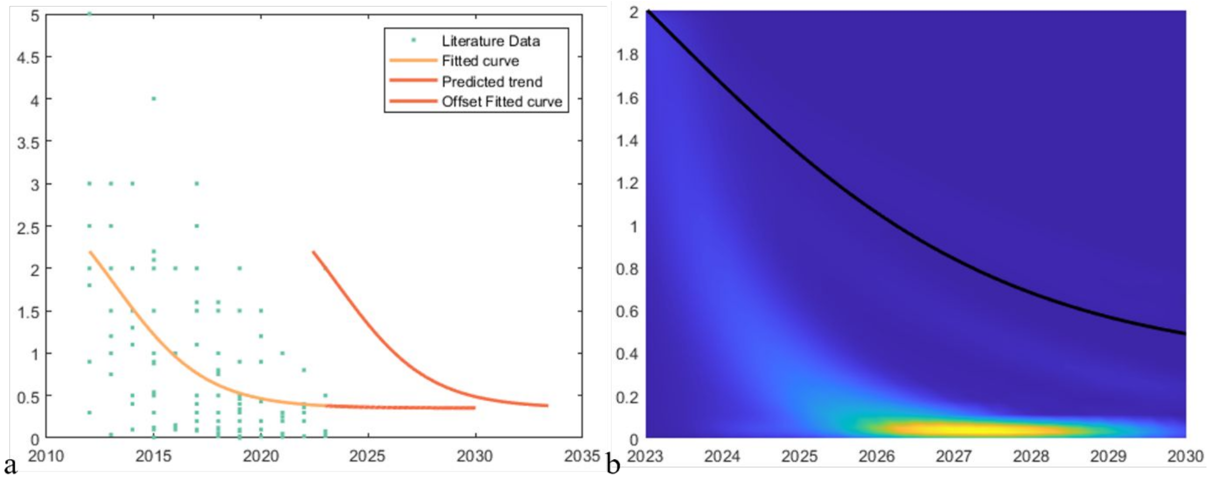

Figure S6: a) Iridium Loading data with prediction and offset b) Heat map of likely technological development routes under emergency development measures.

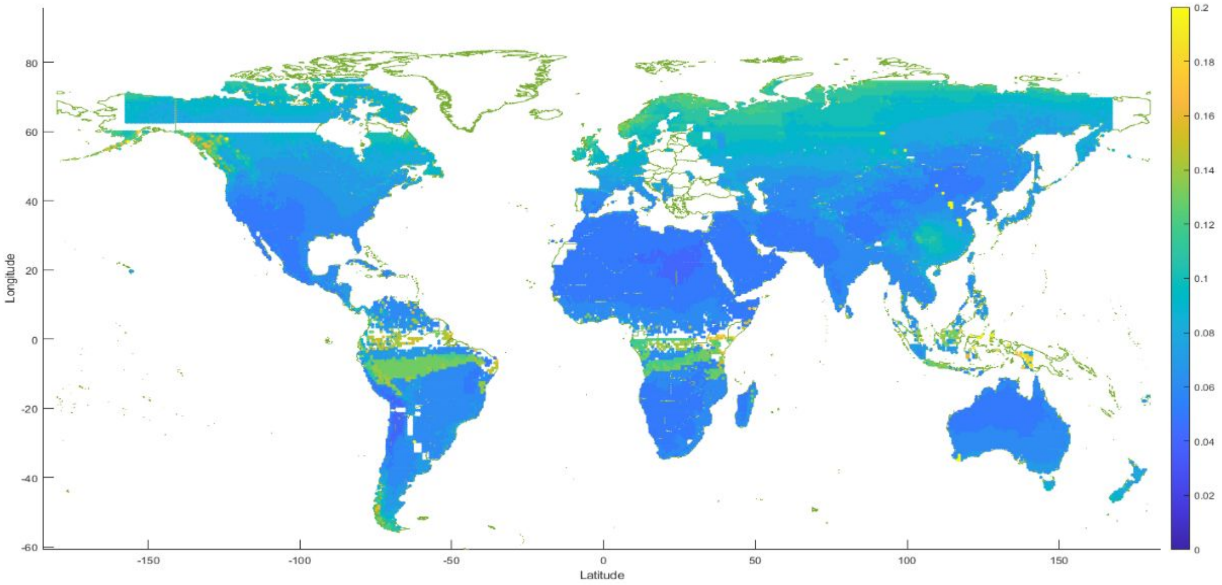

Figure S7. Global solar LCOE values.

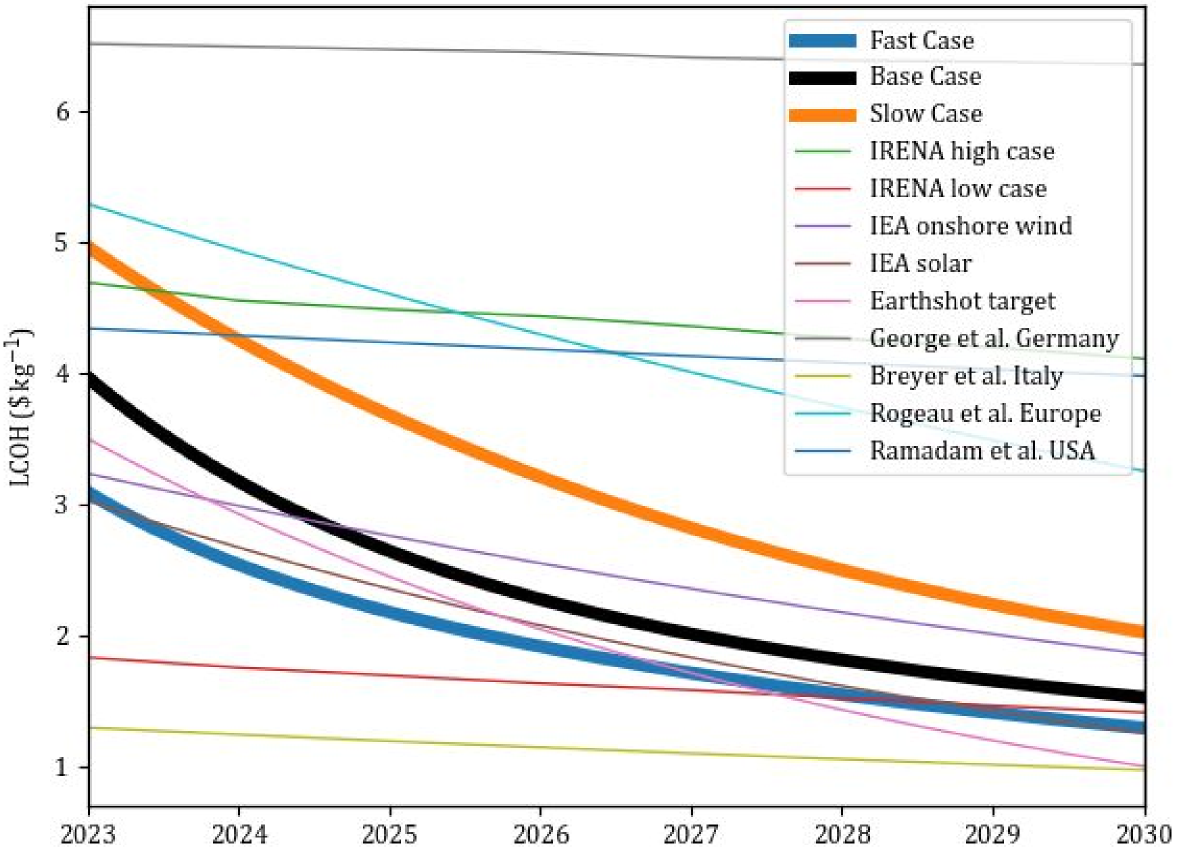

Figure S8. Deployment cases and literature comparison.

Table 1: Base Scenario Parameter Inputs

Parameter	Value
Temperature	80°C
Pressure	30 Bar
Stack Size	2 MW
MEA Lifetime	10000 hours
PTL and Plates Lifetime	20000 hours
Membrane Cost	\$1000 m^{-2}
Iridium Cost	\$158 g^{-1}
Platinum Cost	\$28.5 g^{-1}
Current Density	1 A cm^{-2}

Methodology

Economic Model

The levelised cost of hydrogen (LCOH) is calculated using a combined economic and voltametric model which takes the physical parameters of an idealised proton exchange membrane (PEM) electrolyser to generate operating information about the cell. The economic and voltametric model is defined in equations below.

NPV

The economic model is designed to calculate an NPV, using the yearly cash flow, adjusted for time in years (with a 20 year assumed lifetime), t , and a weighted average cost of capital ($WACC$) of 7.5%. This $WACC$ indicates a moderate risk[S1]. The NPV is dependent on the sale price of hydrogen, which is adjusted to give an NPV value of 0. An NPV of 0 indicates the lowest price the hydrogen could be produced for, and the process still be economically viable.

$$\mathrm{NPV} = \sum \frac{\mathrm{CF_t}}{(1 + \mathrm{WACC})^t} (1)$$

The cash flow is comprised of the net earnings less the depreciation, both in \$ year^{-1}.

$$\mathrm{CF} = [\text{net earnings} - \text{depreciation}] (2)$$

The cash flow includes a year zero where the total capital and working capital expenditures are treated as the yearly outgoings.

Net earnings are the profit adjusted for tax by removing the depreciation, where the profit is the income less outgoings (\$ day^{-1}) multiplied by the working days per year, DPY, assumed to be 350.

$$\text{net earnings} = (\text{Profit} + \text{depreciation}) * (1 - \text{tax}) (3)$$

$$\text{Profit} = \mathrm{DPY} * \ (\text{Income} - (4)$$

The depreciation is calculated using the straight-line method whereby the total capital expenditure, $\mathrm{CAPEX_{tot}}$ (\$) is divided by the lifetime of the plant, in this case 20 years.

$$\text{depreciation} = \frac{-\mathrm{CAPEX_{tot}}}{20} (5)$$

Income

The income is calculated from the mass flowrate of hydrogen produced, $\dot{m}_{H_2}$ (kg day^{-1}), minus losses, $\mathrm{loss_{H_2}}$, and then multiplied by the hydrogen sale price per unit mass, p_{H_2} (\$ kg^{-1}).

$$\text{Income} = \left(\dot{m}_{H_2} - \text{loss}_{H_2}\right) * p_{H_2} \quad (6)$$

The flowrate of hydrogen production can be calculated using the total current, I (A), Faradaic efficiency, FE (%), molecular weight of hydrogen, MW_{H_2} (kg mol^{-1}), number of electrons per mol of product, n_e, and Faraday's constant, F (C s^{-1}). The total current is calculated from the voltametric model and the faradaic efficiency is assume at 97%. The remaining parameters in equation 7 are fundamental to the reaction.

$$\dot{m}_{H_2} = \frac{FE\ MW_{H_2} I}{n_e F} \quad (7)$$

The hydrogen losses are dependent on molecular flux across the membrane, Q_{H_2} (mol m^{-2} s^{-1}), the molecular weight, and the membrane area being considered, A (m^2).

$$\text{loss}_{H_2} = Q_{H_2} MW_{H_2} \quad (8)$$

Molar flux is defined in Eq. 9, where C_{H_2}& C_l are the molar density of hydrogen and water (mol m^{-3}), n_p is the electro-osmotic coefficient (-), Γ_{H_2} is the water solubility of hydrogen (mol mol^{-1}), $D_{H_2}$is the diffusivity of hydrogen in solid polymer electrolyte (m^2 s^{-1}), δ_{mem} is the membrane thickness (m), and T is the temperature (K).

$$Q_{H_2} = \frac{C_{H_2} n_p \Gamma_{H_2} I}{C_l F} \frac{1}{EXP\left[\frac{n_p \Gamma_{H_2} I}{C_l D_{H_2} F} \delta_{mem}\right] - 1} \quad (9)$$

$$n_p = 2.3 + 0.0212\ (T - 80) \quad (10)$$

$$D_{H_2} = 5.65 \times 10^{-8} EXP\left(-\frac{2100}{T}\right) \quad (11)$$

where equations 9-11 are derived in Grigoriev, 2010[S2].

Outgoings

Outgoings represents the negative cash flow from expenses such as utilities and replacement cell parts. These expenses are collectively referred to as operating expenditure or $\mathrm{OPEX_i}$ ($ day^{-1}) where i represents the electricity, elec, the membrane-electrode assembly replacement, MEA, the plates and porous transport layer replacement, plates & PTL, process water purchase, water, and maintenance and compressor costs, main & comp.

$$\mathrm{Outgoings} = \sum \mathrm{OPEX_i} \quad (12)$$

$$\mathrm{i} = [\,\mathrm{elec}, \quad \mathrm{MEA}, \quad \mathrm{plates}, \quad \mathrm{PTL}, \quad \mathrm{water}, \quad \mathrm{main}, \quad \mathrm{comp}] \quad (13)$$

Therefore, the $\mathrm{OPEX_{elec}}$ is simply the daily electrical cost, calculated from the cell current and voltage, E_{cell} (V), multiplied by the price of electricity $\mathrm{p_{elec}}$ ($ kWh^{-1}).

$$\mathrm{OPEX_{elec}} = \mathrm{p_{elec}} E_{cell} \quad (14)$$

$$\mathrm{OPEX_{MEA}} = (\mathrm{p_{membrane}} + \mathrm{p_{catalyst}}) \frac{\mathrm{A}}{\mathrm{life_{MEA}}} \quad (15)$$

The $\mathrm{OPEX_{MEA}}$ is composed of the membrane and catalyst price per unit area, $\mathrm{p_{membrane}}$ & $\mathrm{p_{catalyst}}$ ($ m^{-2}), multiplied by the cell area considered divided by the lifetime of the MEA, $\mathrm{life_{MEA}}$ (day). The catalyst price is dependent on loading. $\mathrm{OPEX_{plates}}$ and $\mathrm{OPEX_{PTL}}$ are calculated similarly to $\mathrm{OPEX_{MEA}}$.

$$\mathrm{OPEX_{plates}} = \frac{\mathrm{p_{plates}A}}{\mathrm{life_{plates}}} \quad (16)$$

$$\mathrm{OPEX_{PTL}} = \frac{\mathrm{p_{PTL}A}}{\mathrm{life_{PTL}}} \quad (17)$$

The $\mathrm{OPEX_{water}}$ is the product of the purchase price of water, $\mathrm{p_{water}}$ ($ kg^{-1}), and the mass flow rate of water, $\dot{\mathrm{m}}_{\mathrm{water}}$ (kg s^{-1}), which in turn is calculated as a required ratio for the hydrogen production rate.

$$\mathrm{OPEX_{water}} = \mathrm{p_{water}}\dot{\mathrm{m}}_{\mathrm{water}} (18)$$

$$\dot{\mathrm{m}}_{\mathrm{water}} = \dot{\mathrm{m}}_{\mathrm{H_2}} \frac{\mathrm{MW_{water}}}{\mathrm{MW_{H_2}} Xr} (19)$$

The $\mathrm{OPEX_{main}}$ is assumed to be 2.5% of the total capital expenditure, $\mathrm{CAPEX_{tot}}$ ($), per year.

$$\mathrm{OPEX_{main}} = \mathrm{CAPEX_{tot}} \times 2.5\%\ \mathrm{year^{-1}} (20)$$

The $\mathrm{OPEX_{comp}}$ is the electrical cost of operating the compressor, calculated by multiplying the compressor electrical power, $\mathrm{Power_{comp}}$ (kW), by the price of electricity.

$$\mathrm{OPEX_{comp}} = \mathrm{Power_{comp}} \mathrm{p_{elec}} (21)$$

The compressor power is derived from Khan et al.[S3], and composed of the number of stages, N (-), the ratio of specific heats, Cr (-), the compressibility factor, z (-), the isentropic efficiency, η_{isen} (-), inlet temperature, $\mathrm{T_{in}}$ (K), the molar gas flowrate, q_m (mol s^{-1}), the universal gas constant, R (J mol^{-1} K^{-1}), the pressure in and out, $\mathrm{P_{out}}$ & $\mathrm{P_{in}}$ (Pa), and the compression ratio, CR (-).

$$\mathrm{Power_{comp}} = \mathrm{N}\left(\frac{\mathrm{Cr}}{\mathrm{Cr}-1}\right)\frac{\mathrm{z}}{\eta_{\mathrm{isen}}}\mathrm{T_{in}}\, q_m\, \mathrm{R}\left(\frac{\mathrm{P_{out}}}{\mathrm{P_{in}}}\right)^{\left(\frac{\mathrm{Cr}-1}{\mathrm{N}*\mathrm{Cr}}-1\right)} \quad (22)$$

$$\mathrm{N} = \frac{\log\left(\frac{\mathrm{P_{out}}}{\mathrm{P_{in}}}\right)}{\log \mathrm{CR}} (23)$$

CAPEX

The capital expenditure, $\mathrm{CAPEX_{tot}}$ ($), is comprised of the capital cost of the cell parts, compressor, and total plant cost, cell, comp, plant, all in $.

$$\text{CAPEX}_{tot} = \sum \text{CAPEX}_\text{i} (23)$$

$$\text{i} = [\, cell, \quad BOC, \quad BOP,] (24)$$

The $\text{CAPEX}_{\text{cell}}$ is composed of each of the cell material costs, $\text{p}_{\text{membrane}}$, $\text{p}_{\text{catalyst}}$, p_{PTL}, p_{plates} all in \$ m^{-2}, multiplied by the considered cell area.

$$\text{CAPEX}_{cell} = (\text{p}_{membrane} + \text{p}_{catalyst} + \text{p}_{PTL} + \text{p}_{plates}) (25)$$

Balance of cell and plant

The balance of cell and plant are defined separately from the rest of the plant capital costs and do not depend on cell CAPEX so that if the cell materials change, the balance of plant will remain independent.

The $\text{CAPEX}_{\text{BOC}}$ is the balance of cell components, endplates, gaskets, and miscellaneous costs, per unit area, multiplied by the cell area.

$$\text{CAPEX}_{BOC} = (\text{p}_{endplates} + \text{p}_{gaskets} + \text{p}_{misc} (26)$$

The $\text{CAPEX}_{\text{BOP}}$ is the sum of the balance of plant units' costs, for the compressor, water purifier, pressure swing adsorption (PSA), thermal management, and piping & control. Thermal management and piping & control are taken as constants, each equal to half the PSA cost.

$$\text{CAPEX}_{BOP} = \text{IF} * \left(\frac{\text{Power}_{comp}}{1000}\right)^{\text{SF}} + \dot{\text{m}}_{water} p_{purifier} + 2 * \text{PSA}_{\text{ref}} \left(\left(\frac{\dot{\text{m}}_{\text{H}_2}}{\dot{\text{m}}_{ref}}\right)^{\text{SF}} + \left(\frac{\dot{\text{m}}_{\text{O}_2}}{\dot{\text{m}}_{ref}}\right)^{\text{SF}} \right) (27)$$

Electrochemical Model

The total cell voltage, E_{cell} (V), is defined as the sum of the thermodynamic, ΔE_{thermo}, resistance, E_{res}, mass transfer & Nernstian conversion (MTNC), E_{MTNC}, and cathodic and anodic overpotentials, $\eta_{cathode}$ & η_{anode}, Voltages, as shown in equation 28[S4].

$$E_{cell} = \Delta E_{thermo} + E_{res} + E_{MTNC} + \eta_{cathode} + \eta_{anode} (28)$$

The thermodynamic voltage is defined as the reversible cell and pH potentials, where the reversible cell voltage is 1.23 V at STP, and the pH potential is defined in Eq. 29 & 30.

$$\Delta E_{thermo} = E_0 + \Delta E_{pH} (29)$$

$$\Delta E_{pH} = 0.0591(pH_{Anode} - pH_{Cathode}) (30)$$

Due to the cathode product being pure H2 which has no pH value, the delta pH value is assumed to be zero.

The resistance voltage is the voltage required to overcome the resistance generated by the membrane, E_{mem}, with the gap and junction resistances, E_{gap} & E_{junc}, being neglected in a PEMWE cell. Membrane resistance is determined by Eq. 32 & 33[S5].

$$E_{res} = E_{mem} + E_{gap} + E_{junc} = E_{mem} (31)$$

The E_{mem} is comprised of the membrane thickness multiplied by the current density, J (A m^{-2}), and divided by the membrane conductivity, σ_{mem} (Ω^{-1} m^{-1}), where λ is the humidification degree (-).

$$E_{mem} = \delta_{mem} \frac{J}{\sigma_{mem}} (32)$$

$$\sigma_{mem} = (0.005139\lambda - 0.00326) EXP\left(1268\left(\frac{1}{303} - \frac{1}{T}\right)\right) (33)$$

The MTNC potential is composed of the conversion and mass transfer, potentials, $\Delta E_{conversion}$ & $\Delta E_{mass\ transfer}$, and overpotentials, $\eta_{cathode,conversion}$ & $\eta_{mass\ transfer}$.

$$E_{MTNC} = \Delta E_{conversion} + \Delta E_{mass\ transfer} + \eta_{cathode,conversion} + \eta_{mass\ transfer} (34)$$

The conversion and mass transfer potentials are calculated as shown in Eq. 35 & 36, using the molar density of hydrogen, oxygen, and water, C_{H_2}, C_{O_2}, C_{H_2O}, all in (mol m^{-3}). They represent the voltages required in order to activate the reaction and drive the mass transport of particles to the active surface of the catalyst materials.

$$\Delta E_{conversion} = -\left(\frac{RT}{n_e F}\right) ln\left(\frac{C_{H_2}{C_{O_2}}^{0.5}}{C_{H_2O}}\right)(35)$$

$$\Delta E_{mass\ transfer} = \frac{RT}{n_e F} ln\left(1 - \frac{J}{J_{lim}}\right)(36)$$

The limiting current density, J_{lim} (A m^{-2}), represents the physical limitation of reactant to reach the surface reaction sites and hence the theoretical maximum value the current density can reach, and is assumed from literature values.

The electrode overpotentials represent the deviation from the theoretical half-cell potential of the electrochemical reactions and are highly dependent on operating conditions and system configuration, as literature values for reference data vary widely.

The cathode conversion overpotential, $\eta_{cathode,conversion}$, is calculated in Eq. 37 where X_r is the conversion of water to hydrogen (-), TS_i is the Tafel slope for either the cathode or anode (V). Eq. 38 calculates the mass transfer overpotential, where J & J_{lim} are the current density and limiting current density, respectively.

$$\eta_{cathode,conversion} = -TS_{cathode}\, log_{10}(1 - X_r)(37)$$

$$\eta_{mass\ transfer} = -TS_{cathode}\, log_{10}\left(1 - \frac{J}{J_{lim}}\right)(38)$$

The cathode or anode over potential can be calculated from Eq. 39, where $\eta_{i,ref}$ is the anode or cathode reference over potential taken from literature, given at a reference current density $J_{i,ref}$ (A m^{-2}), $\eta_{i,kin}$ is the anode or cathode kinetic overpotential.

$$\eta_i = \eta_{i,ref} + \eta_{i,kin} \qquad i = [cathode, anode] (39)$$

$$\eta_{i,kin} = TS_i log_{10}\left(\frac{J}{J_{i,ref}}\right) (40)$$

Current density

Current density, J_i, can be assumed at a set value, which is useful for comparison between real world data where a set current density and subsequent voltage are often used to determine cell performance, and for the generation of polarisation curves. However, the current density can also be linked to the overpotential from the Butler-Volmer equation, where it becomes a function of physical parameters such as catalyst loading and specific surface area[S6]. The Butler-Volmer equation is defined in equation 41.

$$J_i = J_{0,i}\left[EXP\left(\frac{-\alpha_{R,i}F\eta_i}{RT}\right) - EXP\left(\frac{\alpha_{O,i}F\eta_i}{RT}\right)\right] (41)$$

where $J_{0,i}$ is the exchange current density, and $\alpha_{R,i}$ & $\alpha_{O,i}$ are the activity coefficients.

$$J_{0,i} = J_{0,ref}aL\left(\frac{P_A}{P_{ref}}\right)^{\gamma} EXP\left[-\frac{E_c}{RT}\left(1 - \frac{T}{T_{ref}}\right)\right] (42)$$

The exchange current density is analogous to a rate constant, measured when the electrochemical reactions occur at equilibrium, where there is no net current or overpotential. $J_{0,ref}$ is the reference exchange current density measured at STP (A m^{-2}), a is the catalyst specific surface area (m^2 kg^{-1}), L is the catalyst loading (kg m^{-2}), P_A is the partial pressure of the gas species at either the anode or

cathode (Pa), E_c is the activation energy for the electrode reaction (reduction or oxidation) (J mol^{-1}), and P_{ref} & T_{ref} are the reference pressure and temperature (Pa & K).

Equation 41 is valid for the anode and cathode and can be further simplified to equation 43 & 44[S6].

$$J_{cathode} = J_{0,cathode} EXP\left(\frac{-\alpha_{R,cathode} F \eta_{cathode}}{RT}\right) (43)$$

$$J_{anode} = -J_{0,anode} EXP\left(\frac{\alpha_{O,anode} F \eta_{anode}}{RT}\right) (44)$$

References

(S1) International Renewable Energy Agency. *Green Hydrogen Cost Reduction Scaling up Electrolysers to Meet the 1.5°C Climate Goal*; International Renewable Energy Agency, 2020. https://www.irena.org/-/media/Files/IRENA/Agency/Publication/2020/Dec/IRENA_Green_hydrogen_cost_2020.pdf. (accessed 2022-05-06)

(S2) Grigoriev, S. A.; Kalinnikov, A. A.; Millet, P.; Porembsky, V. I.; Fateev, V. N. Mathematical Modeling of High-Pressure PEM Water Electrolysis. In *Journal of Applied Electrochemistry*; 2010; Vol. 40, 921–932. DOI: 10.1007/s10800-009-0031-z

(S3) Khan, T. O.; Young, M. A.; Mackinnon, C. B.; Layzell, C. B. The Techno-Economics of Hydrogen Compression. *TRANSITION ACCELERATOR TECHNICAL BRIEFS* **2021**, *1*, 1–36.

(S4) Shin, H.; Hansen, K. U.; Jiao, F. Techno-Economic Assessment of Low-Temperature Carbon Dioxide Electrolysis. *Nat Sustain* **2021**, *4* (10), 911–919. DOI: 10.1038/s41893-021-00739-x

(S5) Han, B.; Steen, S. M.; Mo, J.; Zhang, F. Y. Electrochemical Performance Modeling of a Proton Exchange Membrane Electrolyzer Cell for Hydrogen Energy. *Int J Hydrogen Energy* **2015**, *40* (22), 7006–7016. DOI: 10.1016/j.ijhydene.2015.03.164

(S6) Barbir, F. Fuel Cell Electrochemistry. In *PEM Fuel Cells: Theory and Practice*; Elsevier, 2013.
